# Computational Analysis Reveals the Temporal Acquisition of Pathway Alterations during the Evolution of Cancer

**DOI:** 10.3390/cancers14235817

**Published:** 2022-11-25

**Authors:** Johanne Ahrenfeldt, Ditte S. Christensen, Mateo Sokač, Judit Kisistók, Nicholas McGranahan, Nicolai J. Birkbak

**Affiliations:** 1 Department of Clinical Medicine, Aarhus University, 8200 Aarhus, Denmark; 2 Department of Molecular Medicine, Aarhus University Hospital, 8200 Aarhus, Denmark; 3 Department of Clinical Oncology, Aarhus University Hospital, 8200 Aarhus, Denmark; 4 Bioinformatics Research Center, Aarhus University, 8000 Aarhus, Denmark; 5 Cancer Research UK Lung Cancer Centre of Excellence, University College London Cancer Institute, Paul O’Gorman Building, London WC1E 6DD, UK; 6 Cancer Genome Evolution Research Group, University College London Cancer Institute, University College London, London WC1E 6DD, UK

**Keywords:** metastasis, cancer evolution, bioinformatics, cancer biology, cancer genomics

## Abstract

**Simple Summary:**

Early stage primary cancer, where the tumour has not yet spread from its site of origin, is commonly curable through surgery alone. Conversely, metastatic cancer, where the tumour has disseminated to distant sites, is almost invariably lethal and is the main cause of cancer death. Given that primary cancer is often curable, it is likely that the ability to metastasise is acquired late in the development of cancer. To investigate if specific cancer gene mutations or pathways are subject to selection at different time points during the life-history of cancer, we compared genomic data from primary and metastatic cancer and timed the acquisition of genomic alterations. The results presented here support the view that certain events are selected for late in the evolution of cancer. However, we observed no difference in the type or timing of events between primary and metastatic cancer. Taken together, our results suggest that the ability to metastasise is not acquired through evolution solely at a genomic level.

**Abstract:**

Cancer metastasis is the lethal developmental step in cancer, responsible for the majority of cancer deaths. To metastasise, cancer cells must acquire the ability to disseminate systemically and to escape an activated immune response. Here, we endeavoured to investigate if metastatic dissemination reflects acquisition of genomic traits that are selected for. We acquired mutation and copy number data from 8332 tumours representing 19 cancer types acquired from The Cancer Genome Atlas and the Hartwig Medical Foundation. A total of 827,344 non-synonymous mutations across 8332 tumour samples representing 19 cancer types were timed as early or late relative to copy number alterations, and potential driver events were annotated. We found that metastatic cancers had a significantly higher proportion of clonal mutations and a general enrichment of early mutations in p53 and RTK/KRAS pathways. However, while individual pathways demonstrated a clear time-separated preference for specific events, the relative timing did not vary between primary and metastatic cancers. These results indicate that the selective pressure that drives cancer development does not change dramatically between primary and metastatic cancer on a genomic level, and is mainly focused on alterations that increase proliferation.

## 1. Introduction

Metastatic disease is the most common cause of cancer related deaths [1] which is usually considered the last step in evolution of lethal cancer. When diagnosed early, cancers are often curable by surgery or radiotherapy, but once the cancer cells have disseminated to distant organs the disease has become systemic and leave most patients incurable. It is therefore of critical importance to understand the evolutionary process of achieving metastatic potential in a primary tumour in order to improve the treatment of cancer. The majority of our understanding of cancer biology comes from studies investigating primary tumours, and while recently more studies exploring metastatic tumours have been published [2,3,4,5,6,7], many fundamental questions about metastatic cancer biology still remain unanswered [8].

Metastasis is a multi-step process, which is referred to as the metastatic cascade [9,10], and includes the steps from local tissue invasion through intravasation into blood vessels and finally colonisation to a distant organ. This process has been described as highly inefficient, with most cells failing to colonise distant locations [10]. The metastatic process has therefore been suggested to depend on both the primary tumour’s ability to shed tumour cells into the blood circulation and the cancer cells’ ability to survive outside of the primary tumour.

Mutations that promote growth and increase cancer cells fitness, are in the literature referred to as driver mutations [11]. Classical oncogenes and tumour suppressor genes improve cancer cell fitness through either increased cell proliferation, or decreased cell death [12]. However, it remains unknown if specific driver mutations or pathway alterations may be linked to metastatic potential [13]. It is possible that there exist specific phenotypic traits that in the primary tumour increase the risk of dissemination or may even act as gatekeeper events that are required for successful metastatic dissemination [12,13].

In the MET500 project by Robinson et al. more than 500 metastatic cancer samples from 20 different cancer types were analysed [4]. Here, they found an increased tumour mutation burden compared to primary tumour samples from The Cancer Genome Atlas (TCGA), and found that on a gene transcription level the metastatic samples had increased global dysregulation. However, they were unable to identify defining characteristics of metastatic development, neither on gene nor pathway level [4]. Similar results were found by Hartwig Medical Foundation (HMF), where 2520 metastatic tumours were whole genome sequenced and analysed [3]. Here, they reported a lack of metastasis-specific driver mutations, again indicating limited metastasis specific evolution. Likewise, a follow-up study by HMF, with paired samples from 250 patients, where the focus was on clinically relevant genomic biomarkers, de Haar and colleagues found full concordance between paired biopsies for 99% of all patients. No single mutation or genomic event has been found to be the basis for metastatic potential, and to this day the metastatic gatekeeper event remains a hypothesis.

More recently, genomic data from unpaired primary and metastatic tumours have been analysed from large cohorts using gene panels. In a study by Nguyen and colleagues, the authors reported a correlation between chromosomal instability and higher metastatic burden [5]. They also observed that specific genomic alterations and signalling pathways are enriched in metastatic samples, but in their gene panel no differences was observed between the type of alterations reported, and the timing of individual events were not performed. In similar work published by us [2], we presented an analysis of 174 common cancer genes based on the GENIE dataset (Genomics Evidence Neoplasia Information Exchange) [14], demonstrating in a larger cohort of more than 40,000 samples how treatment was the dominant evolutionary pressure in metastatic cancer.

With this study we investigate when during the evolution of cancer metastatic po-tential is acquired. Using two publicly available data sets (The Cancer Genome Atlas (TCGA) and the Hartwig Medical Foundation (HMF)), we performed a temporal analysis of more than 4000 primary tumours with whole exome sequencing data, and close to 4000 metastatic tumours with whole genome sequencing data. These datasets were analysed to compare mutations and copy number alterations between the primary and metastatic samples. Furthermore, we divided the mutations into early and late defined by their occurrence relative to copy number alterations in order to explore temporal effects of individual mutation events.

## 2. Materials and Methods

### 2.1. Cohort Overview

**Figure 1:**
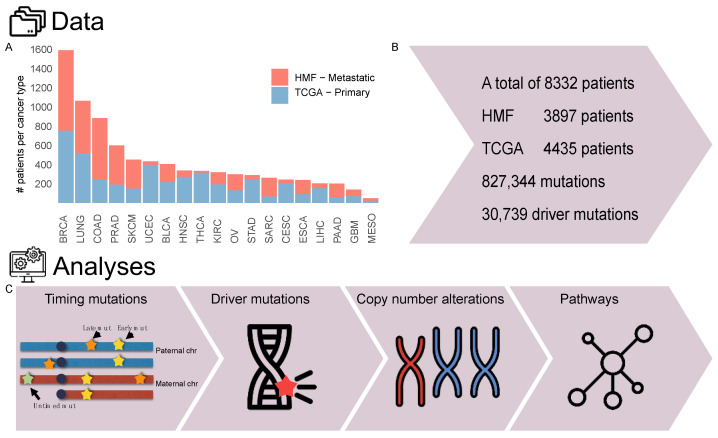
Overview of data and workflow. (**A**) Barplot of number of patients per cancertype. (**B**) Overview of number of patients and number of mutations analysed. (**C**) Workflow showing the outline of the data analysis. First a relative timing of the mutations to the copy number changes was performed. Then, the driver mutations were annotated, and the copy number alterations were analysed, both to summarise them and to annotate driver events. Finally, all driver events were annotated to cancer specific pathways and a pathway enrichment analysis was performed.

To identify metastasis-specific cancer driver events, we acquired two patient cohorts, the Cancer Genome Atlas (TCGA), 4435 samples, representing primary tumours, and the Hartwig Medical Foundation (HMF), 3897 samples, representing metastatic tumours (Figure 1A–C). These cohorts shared 19 cancer types with an average of 225 (range 21–861) metastatic and 251 (range 25–820) primary samples. Cancer type abbreviations are found in Table 1. Hypermutated samples were removed, these were defined as TCGA (whole exome sequenced) samples with more than 1000 mutations, and HMF (whole genome sequenced) samples with more than 60,000 mutations. These cutoffs were defined based on a plot of ascending number of mutations per patient, and the cut off was set approximately where the number of mutations started to rise exponentially (Figure S1).

### 2.2. Relative Timing of Mutations

All mutations were timed relative to copy number events affecting the same genomic location using the method from McGranahan et al., 2015 [15]. The copy number was estimated for each mutation, and then compared to the copy number of the allele. Only regions with at least two copies of the major allele were timed. If the copy number of the mutation was 1 then it was considered late, and if it was above 1 it was considered early. Furthermore, if a mutation was subclonal, it was considered late. To avoid gender bias, only autosomes were used in the analysis.

The Cancer Cell Fraction (CCF) represents the fraction of cancer cells carrying a given mutation. To determine CCF, variant allele frequency (VAF) is integrated with tumour purity and the local copy number, as described [15], according to the formula below:

CCF = VAF × 1/Purity × ((Purity × CN_t_) + CN_n_ * (1 − Purity)), (1)

Here, CN_t_ is the mutation copy number, and CN_n_ is the diploid copy number state.

### 2.3. Annotation of Driver Events

For both TCGA and HMF all somatic mutations were annotated by ANNOVAR [16] with hg19 as the reference genome. Driver mutations were defined as frameshift indels in tumour suppressor genes (TSG) and Non-frameshift indels in oncogenes, with an occurrence in the COSMIC v90 [17] database of at least 3 times. Additionally, defined as driver mutations were deleterious variants in TSGs, either predicted “deleterious” by SIFT, “probably damaging” by PolyPhen (SIFT [18] and PloyPhen [19] scores mutations by their effect on protein function, and are used to annotate the mutations as pathogenic or deleterious) or if the mutation was a stop gain mutation or splice mutations. Finally, we included any specific mutation which was found more than 10 times in the Cosmic database in the definition of driver mutations. For somatic copy number alterations (SCNA) TSGs with deletions and oncogenes with amplifications were classified as driver events.

To evaluate potential driver events in genes not annotated in the COSMIC cancer gene census, we defined driver events in these genes as variants either predicted “deleterious” by SIFT, “probably damaging” by PolyPhen or if the variant was a stop gain mutation, frameshift deletion or insertion. Finally, any specific variant which was found more than 10 times in the Cosmic database was deemed a likely driver.

### 2.4. Copy Number Alterations

The weighted genome integrity index (wGII) was calculated on the available segmented copy number data, as previously described [20]. The loss of heterozygosity (LOH) was defined as a segment where the minor allele had a copy number of 0 and the major allele had a copy number of 1 or more. A genome was said to have undergone genome doubling (GD) if at least half the genome had a major allele copy number of at least 2.

### 2.5. Enriched and Depleted Genes and Pathways

For the enrichment analyses genes were considered altered if they harboured a driver event, as described above. A two-sided Fisher’s exact test was used to compare primary to metastatic samples, on the number of patients with or without altered genes, per cancer type. False discovery rate (FDR) was used to correct *p*-values and considered significant if the corrected *p*-values were below 0.05. All gene driver event were mapped to the cancer specific pathways from Sanchez-Vega et al. [21]. A similar enrichment analysis as above was performed on a pathway level.

### 2.6. Hotspot Mutations

To identify genomic positions with mutation hot-spots, we counted mutated positions for each cancer type in primary and metastatic patients separately. We then used Fisher’s exact test to determine if a significant enrichment or depletion was found for a specific variant.

### 2.7. Statistical Analysis

All analysis was performed in R version 3.6.3 [22], using Tidyverse [23] and ggpubr [24], scales [25], ggrepel [26] for visualisations. For significance testing Wilcoxon test was used unless otherwise mentioned.

## 3. Results

### 3.1. Metastatic Tumours Have a Higher Number of Driver Mutations

In order to compare the number of driver mutations between primary and metastatic tumours, we defined driver mutations based on pathogenic exonic mutations in cancer genes (Methods, Section 2.3). For the TCGA cohort, we identified 15,349 likely driver mutations from a total of 427,237 non-synonymous mutations from 4435 tumours. Similarly, for the HMF cohort, we identified 15,390 driver mutations from a total of 400,107 non-synonymous mutations from 3897 tumours. Overall, we observed that metastatic tumours harboured a slightly higher mean number of driver mutations (mean TCGA = 3.25, mean HMF = 3.65, *p* value < 2 × 10^−16^) (Figure S2A).

**Figure 2:**
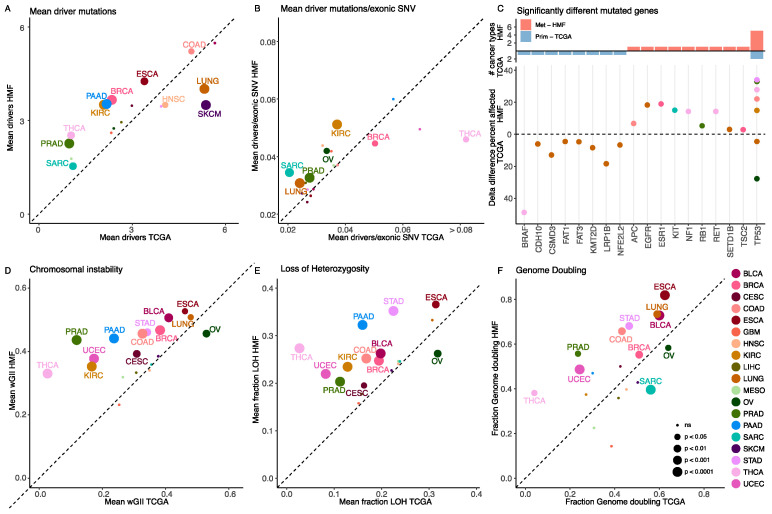
Quantitative analysis of mutations and copy number alterations. (**A**) Scatterplot of the mean number of driver mutations per cancer type in primary vs. metastatic cancer. The size of the bubbles represents the significance of the difference in mean between primary and metastatic samples. (**B**) Scatterplot of the mean number of driver mutations per 100 exonic SNV per cancer type in primary vs. metastatic cancer. The size of the bubbles represents the significance of the difference in mean between primary and metastatic samples. (**C**) The result of the mutation enrichment analysis on gene level is shown in the top panel, with the number of cancertypes the gene is found enriched in. Below the fraction of delta difference in mutations for each of the enriched genes, for each significant cancer type. (**D**) Scatterplot of the mean value of chromosomal instability determined by the wGII score, per cancer type in primary vs. metastatic cancer. The size of the bubbles represents the significance of the difference in mean between primary and metastatic samples. (**E**) Scatterplot of the mean fraction of loss of heterozygosity per cancer type in primary vs. metastatic cancer. The size of the bubbles represents the significance of the difference in mean between primary and metastatic samples. (**F**) Scatterplot of the fraction of patients with genome doubling per cancer type in primary vs. metastatic cancer. The size of the bubbles represents the significance of the difference between the percentage of samples with genome doubling between primary and metastatic, calculated by Fisher’s exact test. For all panels, colours indicate cancer type, as shown in bottom right legend.

To investigate whether a higher number of driver mutations may be a result of increased background mutation burden in metastatic cancer, the number of driver mutations were normalised based on the total number of exonic single nucleotide variants (SNVs) per patient. The number of exonic SNVs was found to be generally higher in metastatic tumours and significantly higher in 7/19 cancer types. However, it was significantly lower in three cancer types (Figure S2B). When we normalised the number of driver mutations by the number of exonic SNVs, we found that the distribution was similar in both cohorts, with a slightly higher mean in the primary cohort (4.1 versus 3.7 driver mutations per 100 exonic SNV, *p* value = 0.048) (Figure S2C). When we compared the number of driver mutations per tumour between primary and metastatic samples within each cancer type, we generally found a higher number of driver mutations per tumour in metastatic samples, which was significantly higher in 8/19 cancer types, whereas there was a significantly higher level in primary cancer for only 3/19 cancer types (Figure 2A). To further explore whether this increase was caused by a general increase in mutations in metastatic tumours, we compared the mean number of drivers per tumour normalised by the exonic SNV count for each cancer type within the two cohorts, and here we found that metastatic tumours harbour significantly more driver mutations in 5/19 cancer types and primary tumours in 2/19 cancer types (Figure 2B). This indicates that the acquisition of driver mutations is not solely driven by an increase in the total number of mutations.

### 3.2. Most Cancer Genes Are Affected by Driver Mutations at the Same Frequency in Primary and Metastatic Cancer

To investigate whether the prevalence of driver mutation events is higher in metastatic samples, we compared the number of driver events between metastatic and primary cancer samples, combining all cancer types. Here, we observed that most driver mutations in specific genes were found at similar frequencies in both primary and metastatic disease. Only 3 genes were found enriched or depleted at frequencies exceeding 5% (TP53 and APC, enriched 15% & 10%, PTEN, depleted 9%, Figure S2D). An additional 682 genes were significantly enriched or depleted in metastatic cancer at frequencies below 5%, with 632 showing less than 1% enrichment or depletion. To investigate if any of the genes showed stronger enrichment or depletion within cancer types, we performed a cancer type specific gene enrichment analysis. Here, we found 18 genes which were significantly enriched or depleted in metastatic cancer. TP53 was the only gene enriched in more than one cancer type, enriched in metastatic cancer in 5 cancer types (KIRC, COAD, TCHA, PRAD and STAD, Figure 2C).

### 3.3. Metastatic Tumours Have a Higher Level of Somatic Copy Number Alterations

To investigate chromosomal instability and the overall level of differences in copy number alterations between metastatic and primary cancer, we determined the weighted genome integrity index (wGII) [20], the fraction of Loss of Heterozygosity (LOH) and the fraction of patients with genome doubling. We found that metastatic tumours had a higher level of copy number alterations relative to primary tumours, with the amount of chromosomal instability, as determined through wGII, was significantly higher in metastatic cancer in 12/19 cancer types. Only in ovarian cancer (OV) did we observe significantly higher levels of wGII in primary tumours (Figure 2D). LOH was also significantly more frequent in metastatic cancer, found increased in 11/19 cancer types. Again, OV stood out as the only cancer type where the frequency of LOH was significantly higher in primary cancer (Figure 2E). The fraction of tumours with genome doubling was significantly higher in metastatic cancer, in 9/19 cancer types. For two cancer types, significantly more tumours were genome doubled in the primary setting (Sarcoma (SARC)) and OV, Figure 2F).

### 3.4. Metastatic Tumours Are More Clonal Than Primary Tumours

**Figure 3:**
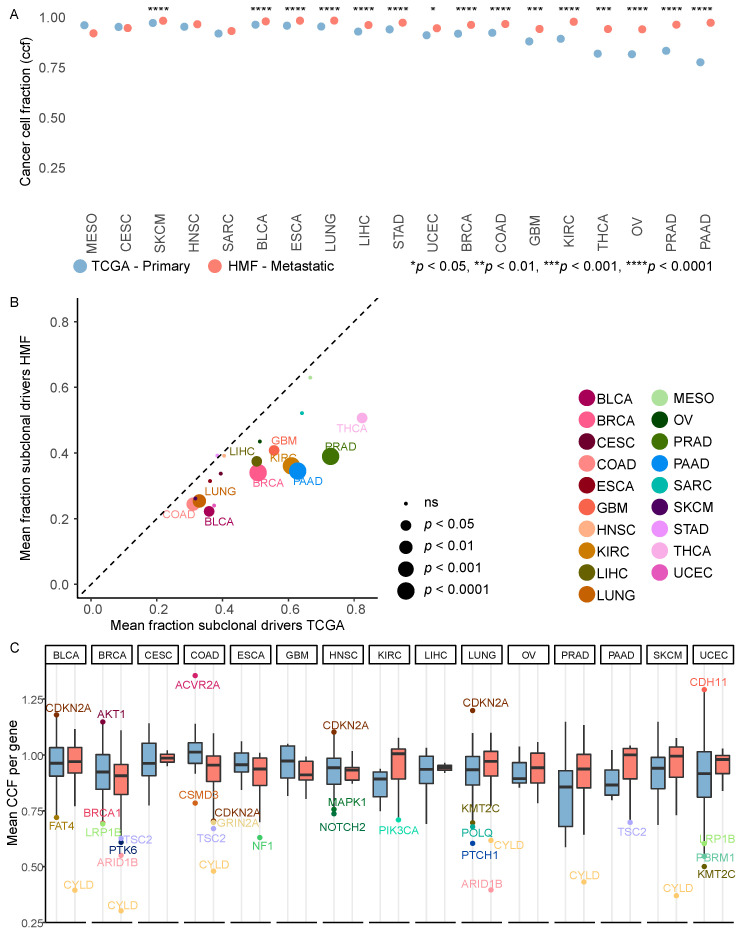
Subclonal mutations. (**A**) Dot plot of the average cancer cell fraction for all mutations in each patient per cancer type, comparing primary and metastatic cancer. (**B**) Scatterplot of the mean fraction of subclonal driver mutations per cancer type comparing primary vs. metastatic cancer. The size of the bubbles represents the significance of the difference in mean between primary and metastatic samples. Colours represent cancer types. (**C**) A boxplot of the mean CCF per gene for all cancer genes with more than 5 hits per cancer type. The outliers are genes with a mean which is more than two standard deviations from the mean of all genes for each cancer type. The lower border of the box represents the 25th percentile, the middle line represents the median, and the upper border represents the 75th percentile.

To investigate the clonality of the mutations and how clonality differs between primary and metastatic cancer, we determined cancer cell fraction (CCF) for all mutations. CCF describes the fraction of cancer cells that carry a given mutation. Thus, for a clonal mutation, a mutation present in all cancer cells in the tumour sample, the CCF value is 1. Likewise, for a subclonal mutation present only in a subset of the cancer cells, the CCF value is below 1. We found that across all cancer types a lower level of subclonal mutations was observed in metastatic tumours compared to primary (Figure 3A). The mean CCF values for all mutations in a sample were significantly higher in metastatic cancer in 15/19 cancer types. This was also true when we investigated driver mutations, here the fraction of subclonal driver mutations was higher in primary tumours across all cancer types, and significantly so in 10/19 cancer types (Figure 3B). We investigated if any genes had more clonal or subclonal driver mutations than average, by calculating the mean CCF per gene, performed for primary and metastatic cancer within the individual cancer types. This was only performed for genes with at least 5 driver mutations per cancer type (Figure 3C). We identified genes with a mean CCF more than two standard deviations from the mean per cancer type, and here we found that *CYLD* had a CCF significantly below average, indicating that it is more subclonal, in metastatic cancer in 6 cancer types (BLCA, BRCA, COAD, LUNG, PRAD and SKCM). When we further explored the frequency and timing of *CYLD* mutations, we found that there were only 12 timed driver mutations of *CYLD* across all primary cancers (4 early, 8 late), whereas there were 98 across metastatic tumours (6 early, 92 late). *ARID1B* also has a CCF below average in two types (BRCA and LUNG), the same goes for *TSC2* (BRCA and PAAD). In primary cancer we found that *CDKN2A* was predominantly clonal in three cancer types (BLCA, HNSC and LUNG). *LRP1B* and *KMT2C* were subclonal in two primary cancers (UCEC and BRCA, UCEC and LUNG, respectively) (Figure 3C).

### 3.5. Driver Mutations Outside Common Cancer Genes

Next, we analysed whether metastatic cancer may select for pathogenic mutations in genes found outside of the COSMIC cancer gene census. For this analysis, we applied a wider definition of driver mutations, including any pathogenic mutations (Methods, Section 2.3). With this definition, each cancer type harboured a high number of mutations, ranging from 5 to 125 mutations per cancer type (Figure S3A). Enrichment analysis identified 47 genes enriched in primary lung cancer, 4 genes enriched in primary melanoma, and 6 genes enriched in metastatic breast cancer (Figure S3B). Notably, lung cancer and melanoma are both carcinogen-induced, and generally harbour a large number of mutations [27]. Given the looser definition of driver mutations used for this analysis, it is possible that gene size rather than gene function may drive the observed enrichment. We observed that the 47 enriched genes in primary lung cancer were on average longer than 84% of all human genes (*p* = 2.9 × 10^−16^), the 4 genes enriched in primary melanoma were on average longer than 97% of all human genes (*p* = 0.0012), while the 6 genes enriched in metastatic breast cancer were on average longer than 94% of all human genes (*p* = 0.00021) (Figure S3C). Taken together, this suggests that the enriched mutations in these genes are likely a reflection of gene size rather than function, as longer genes have a higher probability of being mutated simply because of the length.

### 3.6. Timed Driver Mutations Show Similar Patterns in Primary and Metastatic Tumours

We hypothesised that acquisition of specific driver mutations might give rise to a more aggressive tumour, and essentially function as gate-keepers events. Especially, we wished to explore, whether the metastatic potential and thus a more aggressive tumour occurs at a specific step during cancer evolution. To explore this, all mutations were timed relative to copy number events occurring at the same segment. Here, we found 26% of the mutations were early, 25% late, 18% subclonal in primary tumours. In contrast, we found 26% early, 35% late, 11% subclonal in metastatic tumours. 31% and 29% could not be timed in primary and metastatic tumours, respectively. When we investigated the driver mutations specifically, 65% could be timed in primary tumours. Of these, 49% were early mutations, 25.5% were late and 25.5% were subclonal mutations. Among metastatic tumours, 68 % of the driver mutations could be timed. Of these 58 % were early, 28% were late and 14% were subclonal mutations. More subclonal mutations were found in primary cancer (Figure 3A). Interestingly, there were more early mutations in metastatic cancer, this taken together with there being more timeable mutations in metastatic tumours, may be caused by increased chromosomal instability late in the evolution of cancer, which will result in more early events. To increase power in the analysis, we considered all subclonal mutations as late mutations. To investigate if any genes were enriched in early or late mutations, we performed a gene enrichment analysis of early vs. late mutations for each dataset. We then compared the results for the two datasets, to explore if the timing of the enriched genes were similar. Overall, we only found 23 genes enriched, and the majority of these, 19 genes, were enriched in early mutations. 6 of these genes (*TP53*, *KRAS*, *BRAF*, *VHL*, *PIK3CA*, *AKT1*) were significantly enriched in both primary and metastatic cancers, and only *TP53* and *KRAS* in more than one cancer type, 8 and 2, respectively (Figure S4).

### 3.7. Driver Events in Cancer Specific Pathways Show near Identical Timing in Primary and Metastatic Cancer

**Figure 4:**
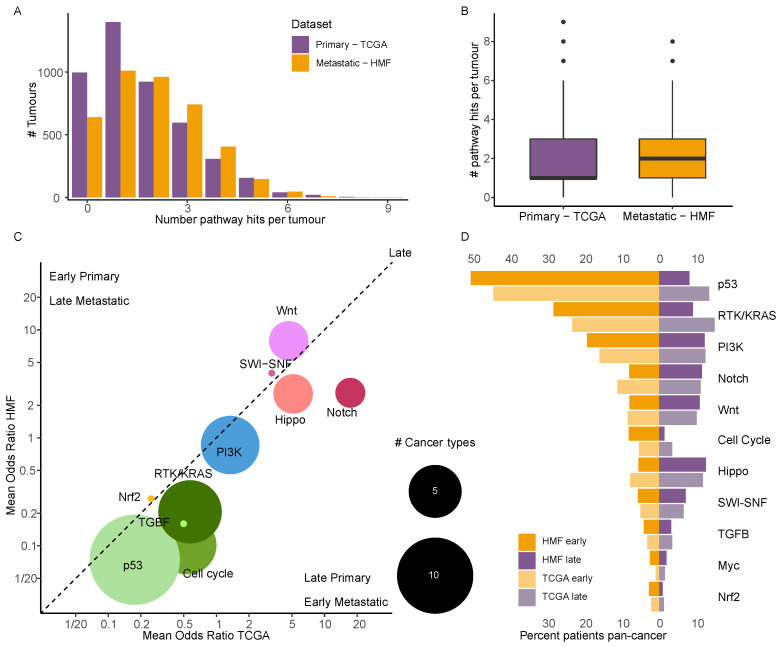
Enriched timed pathways. (**A**) Histogram showing the number of affected cancer pathways by driver mutations. (**B**) Boxplot showing the number of affected specific cancer pathways in primary and metastatic cancer. (**C**) A bubble plot of the mean odds ratio for enrichment of early or late pathway hits in primary vs. metastatic cancer. The size of the bubble shows the number of significant cancer types. (**D**) A mirror plot showing the percentage of patients pan-cancer which have been affected by early and late driver mutations in each of the 11 cancer specific pathways.

Genes and the proteins they code for act in synergy with each other and are connected through signalling pathways. While we only observed limited genomic differences between primary and metastatic cancer, we endeavoured to investigate whether a specific pattern could be observed of which pathways are affected in primary versus metastatic cancer. Therefore, we mapped all driver mutations to established cancer pathways [21]. On a Pan-cancer level we observed that metastatic cancer had a significantly higher number of mutated pathways compared to primary cancer (TCGA: mean = 1.68, median = 1, HMF: mean = 1.99, median = 2, *p*-value < 2 × 10^−16^, Figure 4A,B). We performed a pathway enrichment analysis to explore the timed pathways, and if there were any remarkable difference in timing on a pathway level. As we did for driver genes, we performed an enrichment analysis for early vs. late pathway hits for each dataset, and then we compared the results of the two. When we compared the significantly enriched timed pathways in primary cancer to metastatic cancer, we found that pathway timing was highly concordant, i.e., if a pathway was significantly enriched in early mutations in primary, it was never found significantly enriched for late mutations in metastatic and vice versa (Figure 4C). We observed that the odds ratios for the pathways of two datasets were highly correlated (pearson = 0.79, *p* = 1.82 × 10^−10^). As no shift was observed between primary and metastatic cancer, where events found late in primary cancer became early in metastatic cancer through further selection, this indicates that evolution in the metastatic setting may play a minor role in development of lethal metastatic cancer. The p53 pathway was overall the pathway most enriched in early mutations across cancer types (Primary: 13/19 cancer types, Metastatic: 14/19 cancer types), consistent with a defining role in cancer development. Only CESC, KIRC, MESO, SKCM and THCA were not enriched for early p53 pathway hits, while in PAAD it was only enriched in metastatic tumours. The KRAS and Cell cycle pathways were also highly enriched in early mutations, RTK/KRAS in 7 metastatic and 4 primary cancer types, and Cell cycle in 6 metastatic and 2 primary cancer types (Figure 4C). Conversely, while early pathway hits were quite consistently enriched in p53, RTK/KRAS and Cell cycle pathway genes, late pathway hits affected more pathways. These included Hippo (Primary: 4/19, Metastatic: 3/19) in BRCA and COAD for both and STAD and UCEC for primary and PAAD for metastatic, Notch (Primary: 4/19, Metastatic: 4/19) in COAD and OV for both and UCEC and SKCM in primary and BRAC and PRAD in metastatic, Wnt (Primary: 3/19, Metastatic: 3/19) in BRCA and UCEC for both and STAD for primary and COAD in metastatic (Figure 4C). When we explored the number of times pathways were hit pan-cancer, we found more hits to the three most affected pathways (TP53, RTK/KRAS, PI3K) by early mutations in metastatic cancer, relative to what was observed in primary cancer. However, overall the differences between metastatic and primary cancer were limited and non-significant (Figure 4D), supporting that the timing of pathway hits is concordant (pearson = 0.79, *p* = 1.82 × 10^−10^).

### 3.8. Mutational Hotspots Indicate Treatment Resistance as Driver for Metastatic Evolution

**Figure 5:**
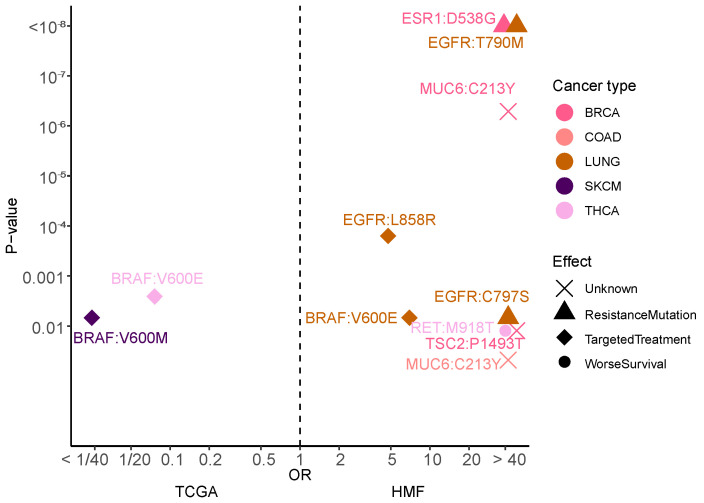
Enrichment of specific variants. A volcano plot showing the variants that are enriched in primary or metastatic cancer. Enrichment determined by Fisher’s exact test. The shape of the dot represents the effect of the mutations based on literature.

Hotspots are defined as specific genomic positions mutated at higher-than-expected frequency across cancer patients. Across the genome, we found 11 variants, which were specifically enriched in metastatic samples (Figure 5). Three variants in the *EGFR* gene were significantly enriched in patients with Non-Small Cell Lung cancer, consistent with acquired resistance to anti-*EGFR* treatment in this cancer type. Likewise, we identified a specific variant in the *ESR1* gene in Breast Cancer, consistent with resistance to anti-hormone treatment. Interestingly, we found significant enrichment of a variant in the *MUC6* gene in Breast Cancer and in Colon cancer, both of which are of unknown effect. In Thyroid cancer we found a variant in *RET* which has previously been linked to worse survival for patients [28]. We also observed two variants that were significantly enriched in primary cancer, both *BRAF* variants with known targeted treatments, most likely a sign that targeted treatment against these two mutations work well, as they do not re-emerge in metastatic cancer. Rather, while metastatic tumours are also enriched in known drivers of aggressive biology such as cell proliferation and chromosomal instability, a key dominant driver of metastatic cancer evolution appears to be anti-cancer therapy.

### 3.9. Loss of Heterozygosity and Driver Mutations Primes for Additional Mutations

**Figure 6:**
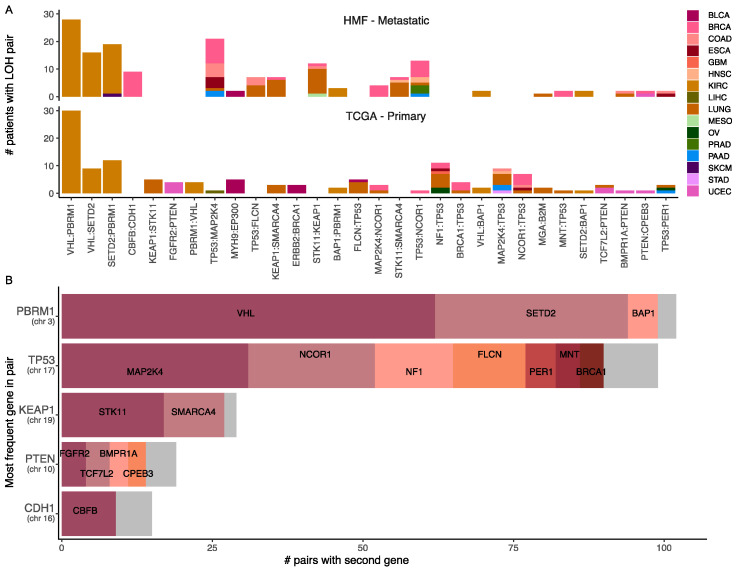
Driver gene pairs in areas of LOH. (**A**) Bar plot showing the pairs of driver genes found in areas of LOH in metastatic (top) and primary (bottom) cancer. (**B**) Stacked barplot showing the partner genes for the five most frequently occurring genes in LOH gene pairs.

We observed a significantly larger frequency of LOH in the metastatic samples. Tumour suppressor genes are commonly affected by a driver mutation that disables a gene copy, followed by a copy number loss event that disables the remaining allele through LOH. However, single copy loss may in this manner provide a first hit to several tumour suppressor genes found within the same genomic region, which may now be limited to a single functioning copy. To investigate if LOH may act as a catalyst for multiple driver mutations acting on tumour suppressor genes, we investigated if areas of LOH with a driver mutation also contained other driver mutations. We did this by exploring pairs of driver mutations on segments with LOH. We found that *TP53* was by far the gene occurring most commonly in pairs across cancer types, found in gene pairs in 12 cancer types. Commonly, *TP53* loss and LOH co-occurred with *MAP2K4* and *NCOR1*, occurring together with *TP53* and LOH 31 and 21 times, respectively, each in 6 different cancer types (Figure 6A,B). This supports that selection for loss of TP53 may result in LOH through single-copy loss, which primes a larger genomic region with effectively a first-hit, leaving it vulnerable to additional inactivating mutations. For KIRC we observed loss of tumour suppressor genes *VHL*, *BAP1*, *PBRM1* and *SETD2* often occuring in pairs (Figure 6A,B). These genes are all found on chromosome 3p, and has previously been reported as co-occurring through sequential loss, where initial mutation and copy number loss of VHL drives further selection of loss of tumour suppressor genes on the same chromosome arm [29]. Some gene pairs were only found in metastatic samples, including *KEAP1*, *SMARCA4*, *STK11*, all found together on chromosome 19, which occurred together in pairs in metastatic BRCA, LUNG, HNSC, COAD and MESO.

## 4. Discussion

With this study we have demonstrated how the relative timing of mutations can give additional information on the mutation patterns of primary and metastatic tumours. Our analysis included data from more than 8000 tumour samples from 19 different cancer types. Cancer driver mutations classically affect tumour suppressor genes and oncogenes, and drive the cancer phenotype through selection for increased proliferation, longevity, chromosomal instability and immune escape [30]. Consistent with previous work we found that metastatic tumours harboured more cancer driver mutations and increased levels of chromosomal instability [5,31], including higher wGII scores, increased fraction of LOH and higher genome doubling rates (Figure 2). However, when we corrected the number of driver mutations by the total number of exonic SNVs, we only found significantly higher levels in metastatic cancer in 5/19 cancer types. We further investigated whether the acquisition of metastatic potential may be driven by mutations acquired in genes outside of established cancer genes (Figure S2), yet we found no evidence of mutations in specific genes enriched in metastatic cancer. Taken together, this supports that the continued acquisition of cancer driver mutations alone play a minor role in the development of traits supporting metastatic dissemination [2].

Previous studies have shown that primary tumours are commonly heterogeneous across cancer types [32]. Tissue biopsies may sample heterogeneous tissue containing multiple subclones, which contains both clonal and subclonal mutations and driver events [13]. Monoclonal dissemination of metastases would result in a bottleneck, where lineage-specific mutations acquired by the metastasising subclone over time would be unmasked. Consistent with this, we found that metastatic tumours harbour more driver mutations, and more genomic alterations compared to primary tumours, as previously reported [3,4,5,13,33]. Additionally, primary tumours harboured more subclonal mutations relative to metastatic tumours, while metastatic harboured more clonal mutations (Figure 3A,B). While we found higher levels of driver mutations in metastatic tumours, the number of drivers per exonic mutation were more even between primary and metastatic tumours. This indicates that for the majority of tumours no additional canonical driver mutations are required for metastatic transition and thus not selected for. Taken together, the increased mutation burden and increased clonality in metastatic tumours overall supports a monoclonal dissemination pattern, driven by few specific cancer driver events that are already selected for in the primary tumour.

While subclonal alterations were less common in metastatic tumours, we did observe subclonal alterations. In our work we calculated the mean CCF per gene per cancer type, and here we identified 33 genes with a mean CCF that deviated more than 2 standard deviations from the mean value of each cancer type. The most commonly subclonal gene found in metastatic tumours was *CYLD*, found in 6 different cancer types (BLCA, BRCA, COAD, LUNG, PRAD and SKCM). *CYLD* is a tumour suppressor gene involved in regulation of several proliferation-associated pathways, including NF-κB, Wnt, Notch and TGF-β, potentially suggesting that metastatic tumour heterogeneity driven by further adaptation to increased proliferative phenotypes does occur, though less ubiquitous than the heterogeneity observed in primary tumours.

From the gene enrichment analysis we found that *ESR1* was significantly enriched in metastatic relative to primary breast cancer. This result is almost certainly induced by treatment, as ER+ breast cancer patients as standard, are treated with adjuvant anti-hormone therapy. We also observed specific enrichment of two other known resistance mutations *EGFR* T790M and C797S in non-small cell lung cancer, again this is almost certainly induced by treatment with *EGFR* inhibitors [34]. Our findings demonstrate how treatment changes the evolutionary pressures on cancer, inducing selection for resistance-associated drivers and clonal bottlenecking in metastatic cancer. In primary cancer we found two enriched variants of *BRAF*, in melanoma and thyroid cancer, these are both variants that can be targeted by treatment [35] and therefore likely are selected against in development of metastatic cancer.

It is hypothesised that acquisition of the metastatic cancer phenotypes is a late-stage event, where cancer cells acquire the capacity for systemic colonisation of distant sites [10], potentially facilitated through late driver mutations affecting relevant pathways. To investigate this, we analysed the timing of pathway alterations in metastatic and primary cancer (Figure 4). We did not find any additional driver mutations in the known cancer genes. Neither did we find that metastasis is driven by a certain order of events. Rather, we found that the relative timing of driver events in known cancer pathways are very similar between primary and metastatic tumours. Indeed, so similar that when we plot the odds ratio of the pathways being significantly enriched in late or early driver mutations, in primary vs. metastatic tumours, there is a significant correlation with a Pearson coefficient of 0.79. Additionally, in no cancer type is a late primary event significant in early metastasis, or vice versa (Figure 5).

## 5. Conclusions

Taken together, our results support a model where cancer driver events required for metastatic dissemination likely occur relatively early in the life-history of cancer. However, given that a large fraction of patients remains curable by surgery alone despite harbouring tumours with aggressive cancer mutations, there may be other non-genomic factors that are required for metastatic progression. These could include transcriptomic reprogramming, development of immune-evasion phenotypes through non-genomic mechanisms, or potentially cancer-induced degradation of the host immune response through interaction with the tumour immune microenvironment.

## Figures and Tables

**Table 1:** Cancer type abbreviations from the Cancer Genome Atlas.

Abbreviation	Cancer Type
BLCA	Bladder Urothelial Carcinoma
BRCA	Breast invasive carcinoma
CESC	Cervical squamous cell carcinoma and endocervical adenocarcinoma
COAD	Colon adenocarcinoma
ESCA	Esophageal carcinoma
GBM	Glioblastoma multiforme
HNSC	Head and Neck squamous cell carcinoma
KIRC	Kidney renal clear cell carcinoma
LIHC	Liver hepatocellular carcinoma
LUNG	Non small cell lung cancer
MESO	Mesothelioma
OV	Ovarian serous cystadenocarcinoma
PAAD	Pancreatic adenocarcinoma
PRAD	Prostate adenocarcinoma
SARC	Sarcoma
SKCM	Skin Cutaneous Melanoma
STAD	Stomach adenocarcinoma
THCA	Thyroid carcinoma
UCEC	Uterine Corpus Endometrial Carcinoma

## Data Availability

Not applicable.

## Supplementary Materials

The following supporting information can be downloaded at: https://www.mdpi.com/article/10.3390/cancers14235817/s1, Figure S1. (A) All TCGA patients ordered by total number of mutations (Y-axis), as defined by whole exome sequencing. Black line represents 1000 mutations, and is used to define hypermutated samples. (B) All HMF patients ordered by total number of mutations (Y-axis) as defined by whole genome sequencing. Black line represents 60,000 mutations, and is used to define hypermutated samples. Figure S2. (A) Histogram showing the distribution of driver mutations per tumour. (B) Histogram showing the distribution of driver mutations per 100 exonic SNVs per tumour. (C) A violin plot showing the distribution of Exonic SNVs per patient for each cohort in each cancer type. Arranged by ascending median. (D) A plot showing the delta difference in mutations between primary and metastatic cancers for the 25 most differently mutated genes in each direction. Figure S3. (A) Scatterplot of the mean number of pathogenic mutations outside of common cancer genes, per cancer type in primary vs. metastatic cancer. (B) Volcano plot showing enrichment of non-cancer genes with pathogenic mutations in primary versus metastatic tumours. (C) Density plot showing the distributions of gene lengths for all genes and for cancer driver genes (COSMIC cancer gene census) and three boxplots with the lengths of the genes enriched outside of the usual cancer driver genes, in primary LUNG, primary SKCM and metastatic BRCA. For all panels, colours indicate cancer type, as shown in the right side legend. Figure S4. A bubble plot of the mean odds ratio for enrichment of early or late driver mutations in primary vs. metastatic cancer. The size of the bubble shows the number of significant cancer types and the shape shows if the gene is significant in one or both cohorts.
