# Supplementary figures and images for: Computational Analysis Reveals the Temporal Acquisition of Pathway Alterations during the Evolution of Cancer
Source: Cancers (Basel). 2022 Nov 25;14(23):5817. doi: 10.3390/cancers14235817 (PMC9739002; doi:10.3390/cancers14235817)

**A**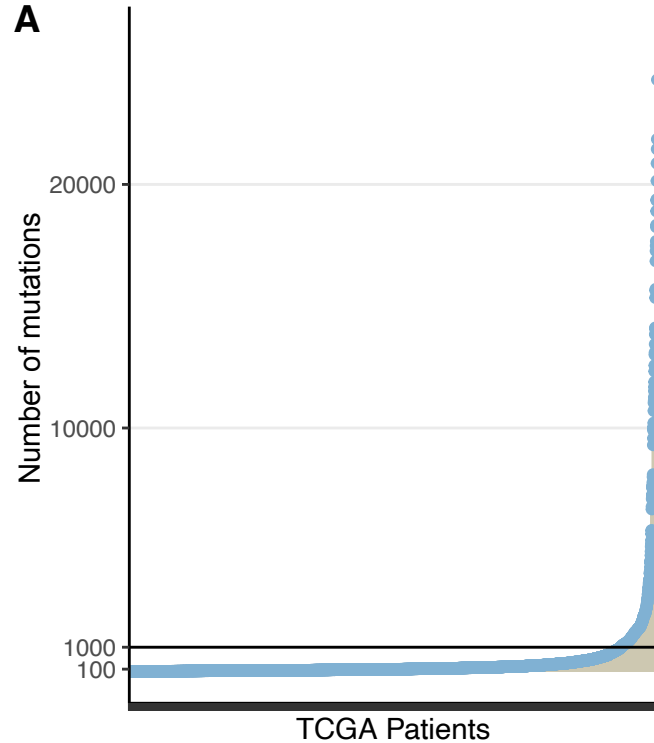**B**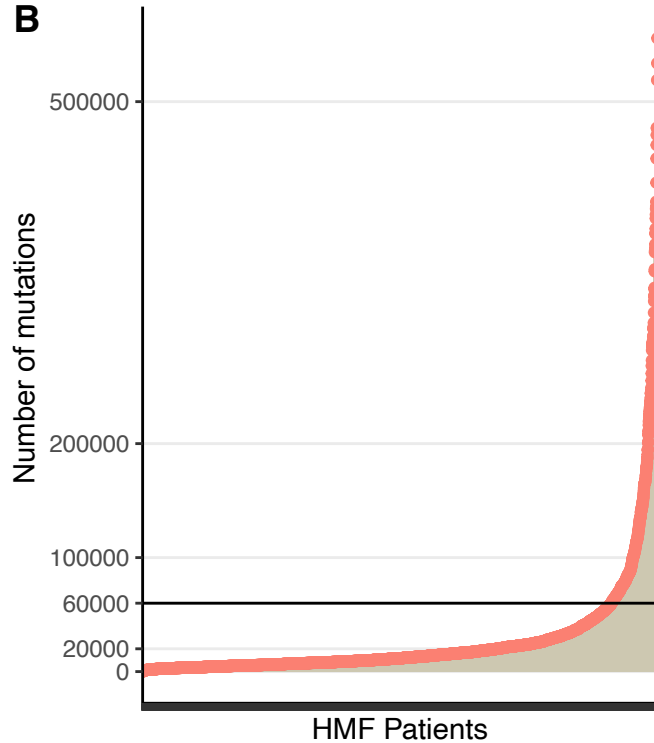

Supplement: Supplementary file 1 [file cancers-14-05817-s001.zip › Supplementary_Figure_S1.pdf]

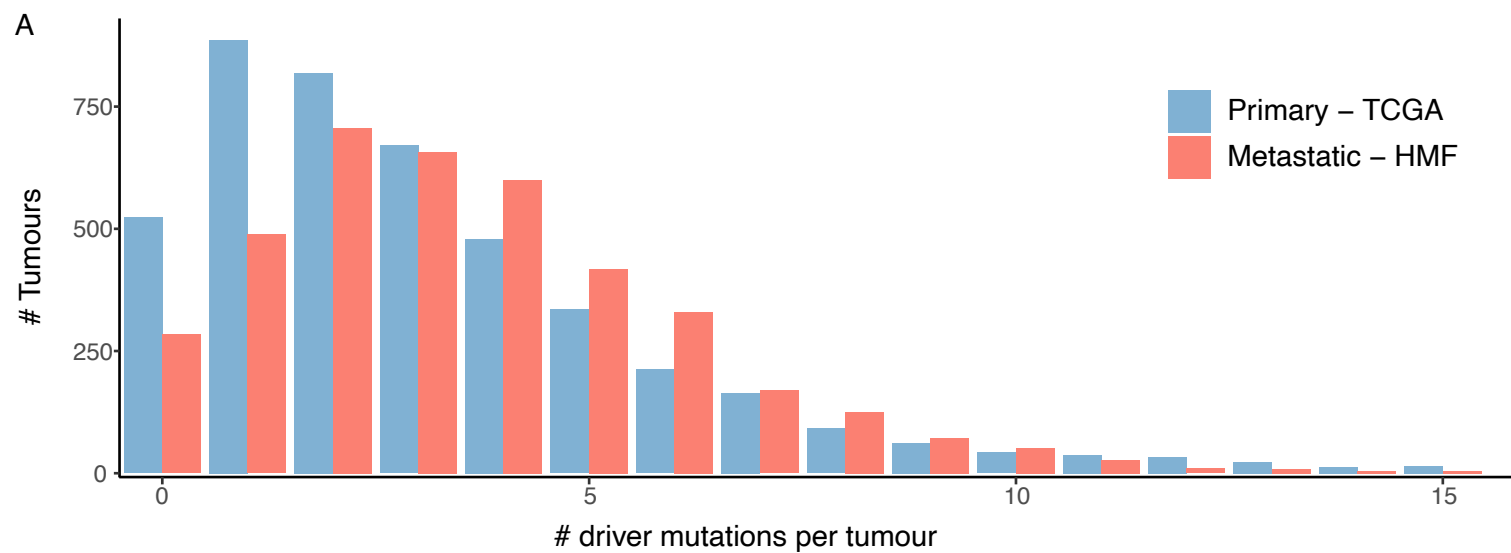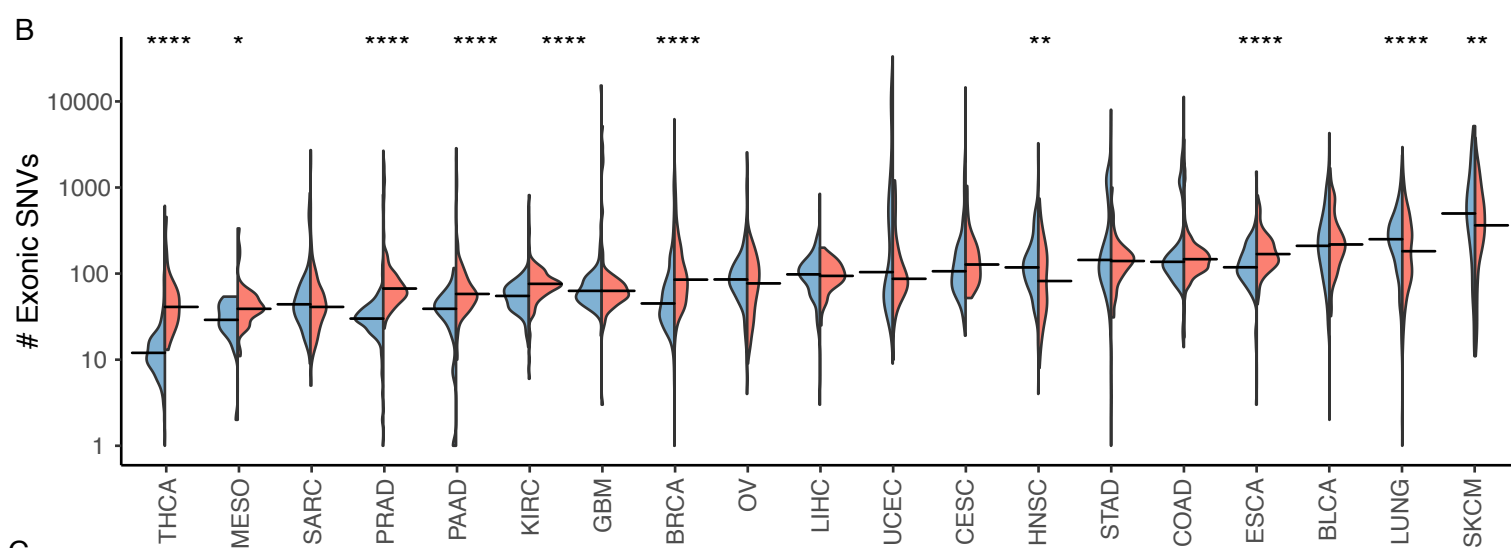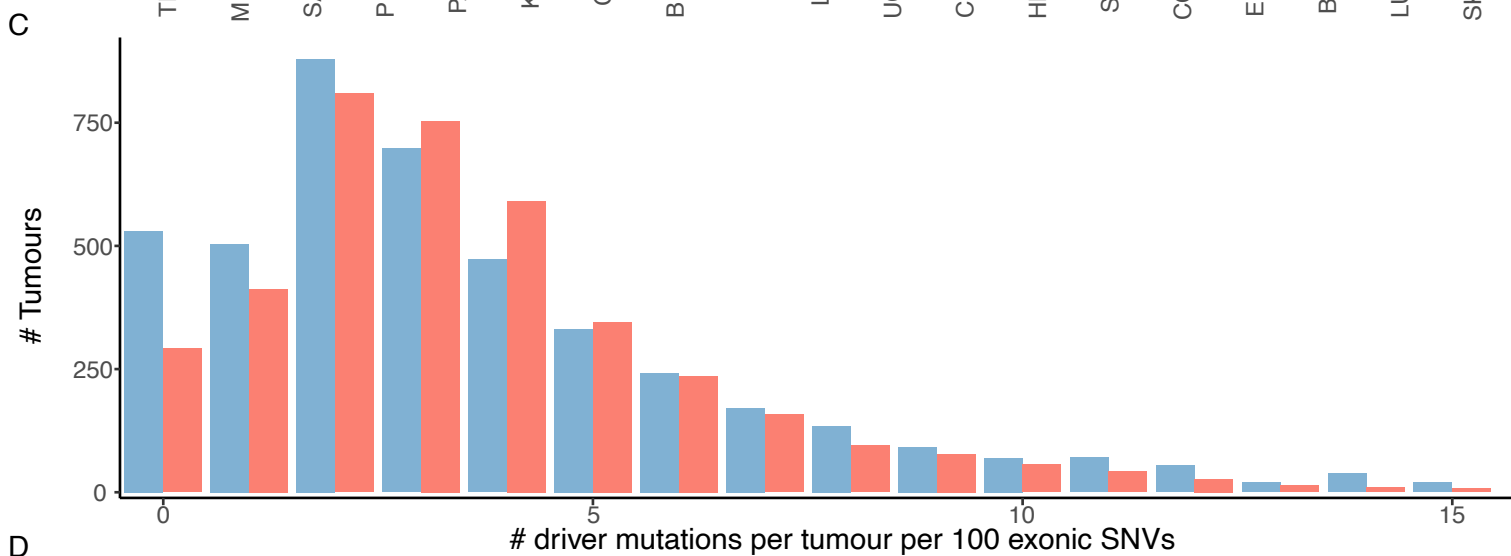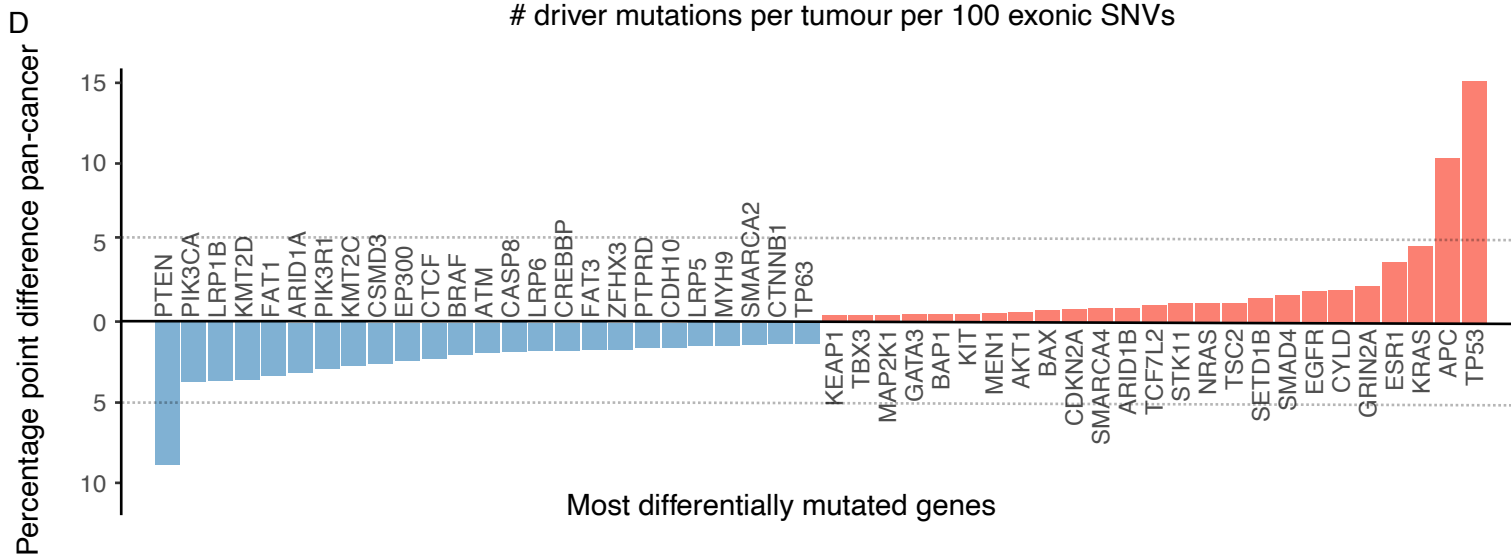

Supplement: Supplementary file 1 [file cancers-14-05817-s001.zip › Supplementary_Figure_S2.pdf]

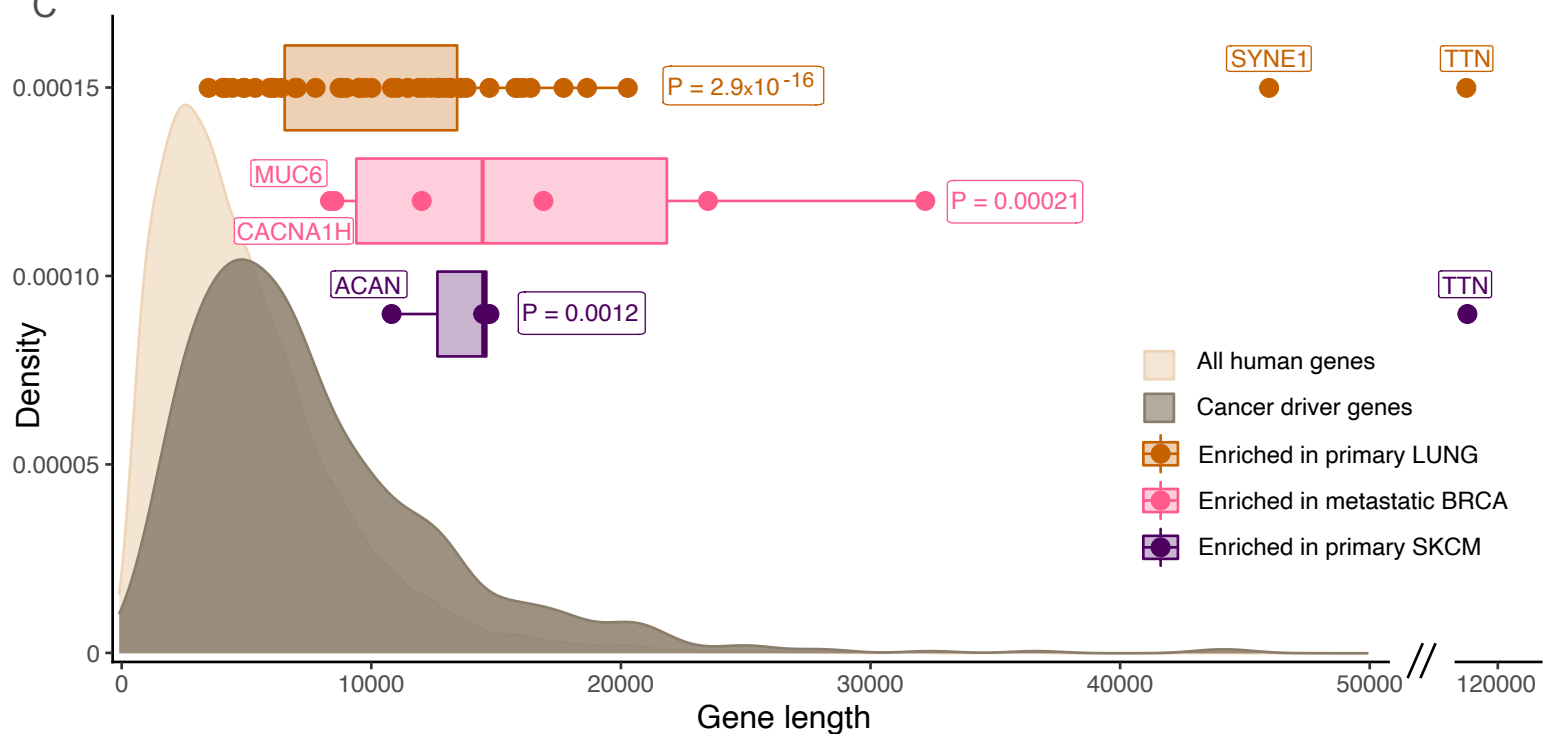

Supplement: Supplementary file 1 [file cancers-14-05817-s001.zip › Supplementary_Figure_S3.pdf]

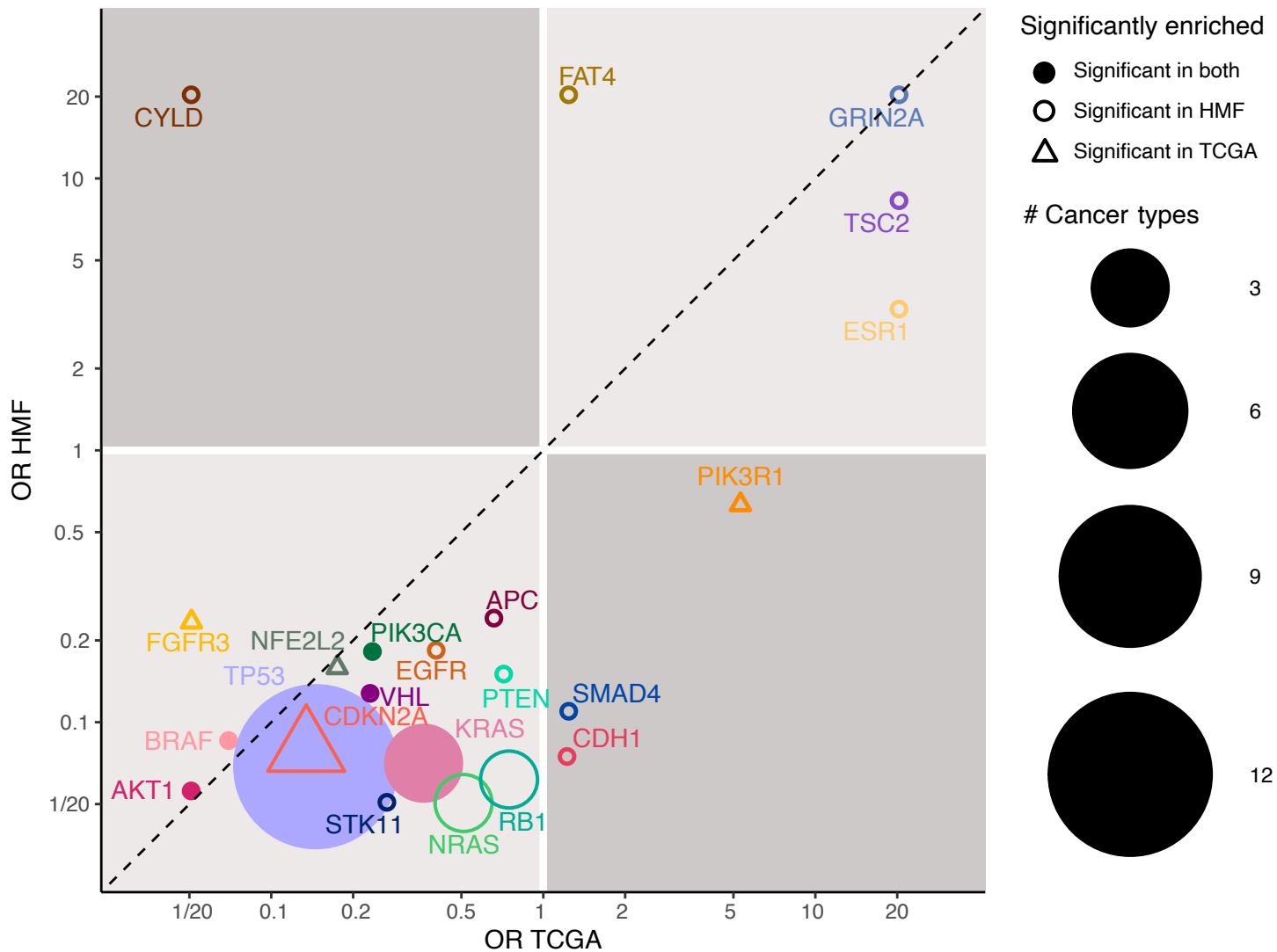

Supplement: Supplementary file 1 [file cancers-14-05817-s001.zip › Supplementary_Figure_S4.pdf]
